# Tuberculosis in the Dog

**Published:** 1897-11

**Authors:** 


TUBERCULOSIS IN THE DOG.—The subject affected was eight years of age, and for five months had suffered with acute enteritis. The animal was very much depressed and very thin, and Dr. Mouguet suspected that it was affected with tuberculosis. Friction by the hand revealed a hypertrophy of the liver and intense sensibility over that region. Auscultation did not indicate any pulmonary lesions, ascites followed, and the animal was destroyed. The liver was very much enlarged, and presented tubercular masses of different sizes, some as large as an apple, and the central masses containing quantities of purulent matter were broken down. The peritoneum and the pleura in the region of the diaphragm were covered with tubercular masses. The lungs were filled with similar tubercles, attaining in size a grain of hempseed. An examination under the microscope developed the bacteria of tubercles as being the cause of these lesions. The use of tuberculin produced a well-marked reaction in the dog.—*Revue Veterinaire.*

